# Analysis of the Vertical Driving Performance of Multiple Connected Pipe-Climbing Microrobots with Magnetic Wheels

**DOI:** 10.3390/mi10080524

**Published:** 2019-08-09

**Authors:** Munehisa Takeda, Isao Shimoyama

**Affiliations:** 1 MEMS System Development Center, Micromachine Center, Tokyo 101-0026, Japan; 2 Toyama Prefectural University, Imizu 939-0398, Japan; 3 Graduate School of Information Science and Technology, The University of Tokyo, Tokyo 113-8656, Japan

**Keywords:** microrobot, magnetic wheel, pipe climbing, driving performance, slip, magnetic attraction, hoop force, multiple connected

## Abstract

In this study, we analyzed the vertical driving performance of multiple connected magnetic wheel-driven microrobots when moving up and down a small cylinder that simulated a pipe. The dynamics of pipe climbing by the magnetic wheel-driven microrobot were analyzed considering the magnetic attraction force and slip; a vertical climbing simulator was developed considering the hoop force and external force from the adjacent microrobots to determine the magnetic attraction force required for multiple connected microrobot pipe climbing. A prototype of an independent vertical climbing microrobot, 5 mm long, 9 mm wide, and 6.5 mm high, and prototypes of 10 microrobots were manufactured to evaluate the vertical driving performance. The usefulness was verified by showing that three driving microrobots can move seven non-driving microrobots comprising 60% of their own weight up and down along a small cylinder.

## 1. Introduction

Mobile robots can considerably expand the applications of robots. Through a reduced size, mobile robots can perform operations that cannot be performed by humans or conventional machines, such as narrow-place operations. Research on autonomous microrobots with autonomous functions is being actively conducted. Many studies have been conducted on autonomous microrobots, such as moving in liquid [1,2,3] and moving in the air [4,5]. Active research and development is being conducted to create the field of wall mobile robots. Wall-climbing robots are expected to find applications in the fields of inspection, testing, construction, cleaning, transportation, and security. For wall climbing, moving and wall adhesion mechanisms are necessary. Crawler, wheeled, legged, and propulsion methods have been developed as moving methods, and suction, magnetic, grasping grippers, thrust force adhesion, and biologically inspired adhesion methods have been developed [6,7,8,9]. Wall-climbing microrobots have been developed for applications in small spaces such as for the inspection of small pipes in power generation plants [10,11,12]. Since configuring a complex mechanism with wall-climbing microrobots is difficult, two methods have mainly been used for adhesion to walls: the biologically inspired adhesion method [13,14] and the magnetic wheel method [10,11,12,15,16]. Greuter et al. developed a 40 mm × 43 mm × 14 mm crawler-type wall-climbing microrobot using silicone rubber [13]. Fischer et al. developed a foldable, magnetic, and wheel-driven wall-climbing robot for inspecting gas turbines [15]. Rochat et al. developed a 28-mm diameter cylindrical wall-climbing microrobot (Cy-mag 3D) with a unique structure that uses magnets [16]. Tang et al. and Zhang et al. developed a magnetic wheel driven omni-directional wall-climbing microrobot (diameter: 26 mm, height: 16.4 mm) using magnetic micromotors [10,11]. This author and colleagues also developed a magnetic wheel-driven microrobot [12].

However, few studies on wall-climbing microrobots have considered the gripping force as well as the slip characteristics of wheels. Although many studies have focused on independent robots for wall climbing [6,7,8,9], studies on wall climbing by connecting multiple robots are limited. Since equipping a microrobot with many functions is difficult given their small size, research on multiple microrobots working in coordination as a group will be needed in the future, and for this, determining the required conditions for the travel of multiple connected microrobots is considered important.

This study reports the results of an analysis of the dynamics of pipe climbing by magnetic wheel-driven microrobots, considering the magnetic attraction force and slip by incorporating the results of the horizontal driving clarified in [17]. We identify the magnetic attraction force necessary for pipe climbing. We also report a vertical climbing simulator for multiple connected microrobots, which considers hoop force and external force from the adjacent microrobots. The results of the vertical driving performance of an independent microrobot and 10 connected microrobots using simulation and prototypes of the microrobots are reported.

## 2. Development of a Vertical Climbing Simulator

### 2.1. Six-Wheeled Model of a Microrobot

**Figure 1:**
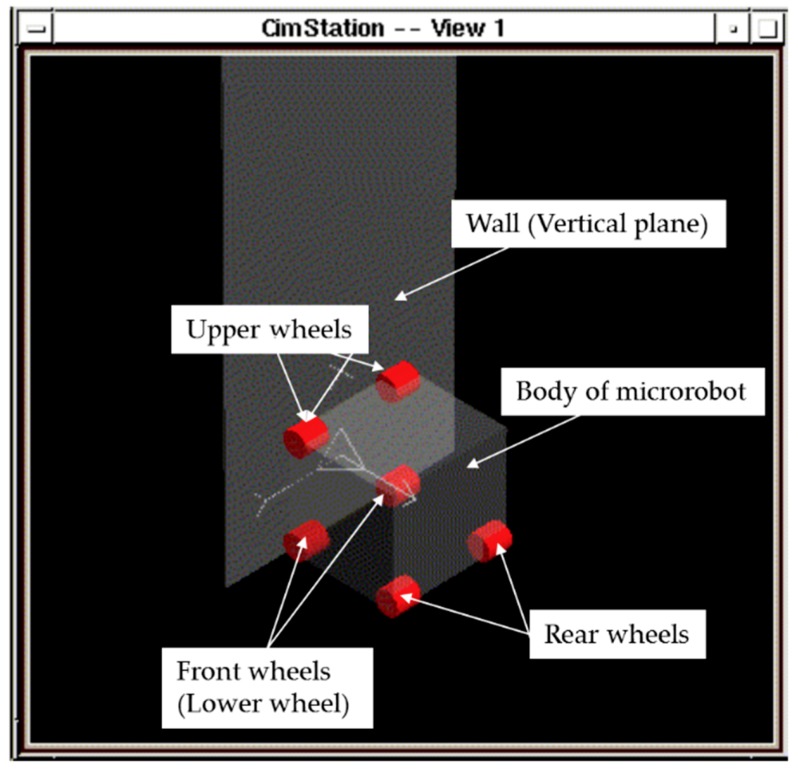
Six-wheeled model of a microrobot. This model has four magnetic wheels in the horizontal plane and four magnetic wheels in the vertical plane. The front wheels (lower wheels) are the driving wheels, and the rear and upper wheels are the driven wheels.

A six-wheeled model of a microrobot that can move in the horizontal and vertical planes is shown in Figure 1. This model has 4 magnetic wheels in the horizontal plane and 4 magnetic wheels in the vertical plane. The front wheels (lower wheels) are the driving wheels, and the rear and upper wheels are the driven wheels. 

**Figure 2:**
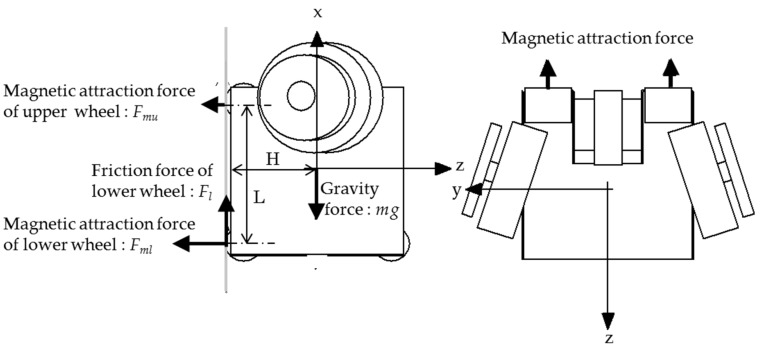
Forces acting on the microrobot when climbing on a wall. *F_l_* is the friction force on the lower wheel, *F_mu_* and *F_ml_* are the magnetic attraction forces of the upper and lower wheels, *m*g is the gravity force, H is the height of the center of gravity, and L is the wheel distance. The magnetic attraction forces in the upper wheels (driven wheels), *F_mu_,* must be actuated to overcome the rotational moment due to its weight in addition to the gripping forces in the lower wheels (driving wheels), *F_l_,* to keep the wheels in movement.

When climbing a wall, as shown in Figure 2, the magnetic attraction forces in the upper wheels (driven wheels) must be actuated to overcome the rotational moment due to its weight, in addition to the gripping forces in the lower wheels (driving wheels) to maintain the movability of the wheels. Since friction forces do not act on the upper wheels, as they are driven wheels and do not slip, Equations (1) and (2) are established as static equilibrium from the balance of force in the vertical direction and balance of the moment around the lower wheels:

(1) $$F_{l}=mg,$$

(2) $$m\mathrm{gH}=F_{mu}L,$$

where *F_l_* is the friction force on the lower wheel, *F_mu_* and *F_ml_* are the magnetic attraction forces of the upper and lower wheels, *m*g is the gravity force, H is the height of the center of gravity, and L is the wheel distance.

Dynamically, acceleration acts in different directions, and slip is also generated during the up and down motion; studies considering these dynamics are required to determine the necessary magnetic attraction force. Therefore, we developed a unique vertical climbing simulator considering slip and magnetic attraction force using a three-dimensional (3D) horizontal travel simulator that considers slip and magnetic attraction force. The simulator was developed using a motion analysis program (SilTools) based on object-oriented language.

### 2.2. Climbing Simulator for Multiple Connected Microrobots

To examine the feasibility of the prototype microrobot system, we developed a climbing simulator for multiple connected microrobots using the specifications of the prototype microrobot. The climbing simulator for multiple connected microrobots simulates the situation where 10 microrobots located around a small cylinder are connected and move up and down. In addition to evaluating the possibility of moving up and down and the driving performance of connected movement by setting the rigidity of the connector spiral spring and magnetic attraction forces of the magnetic wheels, and considering the forces acting between the adjacent microrobots, the specification of the connected climbing microrobot was determined by arbitrarily changing the arrangement of the driving and non-driving microrobots with different specifications and mass around the cylinder.

**Figure 3:**
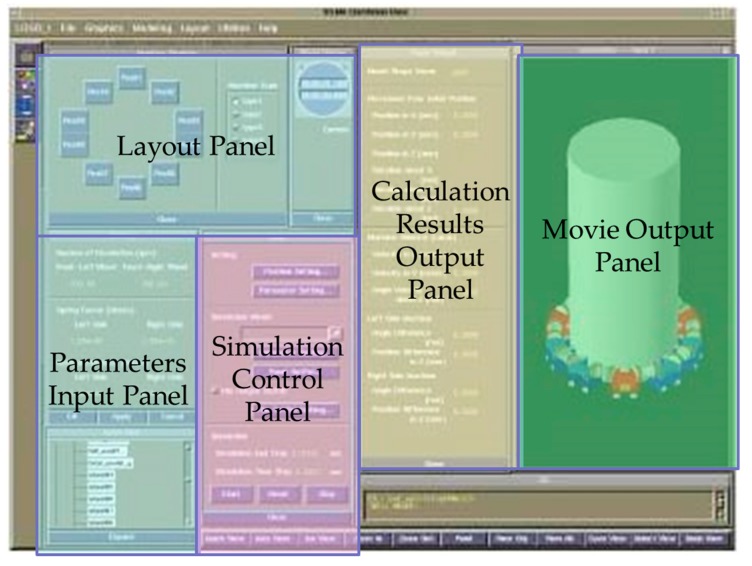
Input/output panel of the vertical climbing simulator for multiple connected microrobots. The left and upper left areas show panels for the input of parameters and layout around a small cylinder. The center left area shows the simulation control panel. The center right area shows the calculation results output panel. The right area shows the movie output panel.

When moving up and down the small cylinder, the connectors are subject to forces from the adjacent microrobots in addition to gravity, driving force, and magnetic attraction force. The movement for the next time step of each microrobot is determined from the equation of motion related to the microrobot coordinate system. The simulation was used to evaluate the separation of connectors and interference with adjacent microrobots and to measure displacements in the vertical direction on the small cylinder. Then, we determined whether movement was possible by connecting 10 microrobots. An example of the input/output panel of the vertical climbing simulator for multiple connected microrobots is shown in Figure 3. The left and upper left areas show panels for the input of parameters and layout around a cylinder. The center left area shows the simulation control panel. The center right area shows the calculation results output panel. The right area shows the movie output panel.

The details of the vertical climbing simulator for multiple connected microrobots are described below.

#### 2.2.1. Layout of Microrobots around a Small Cylinder

**Figure 4:**
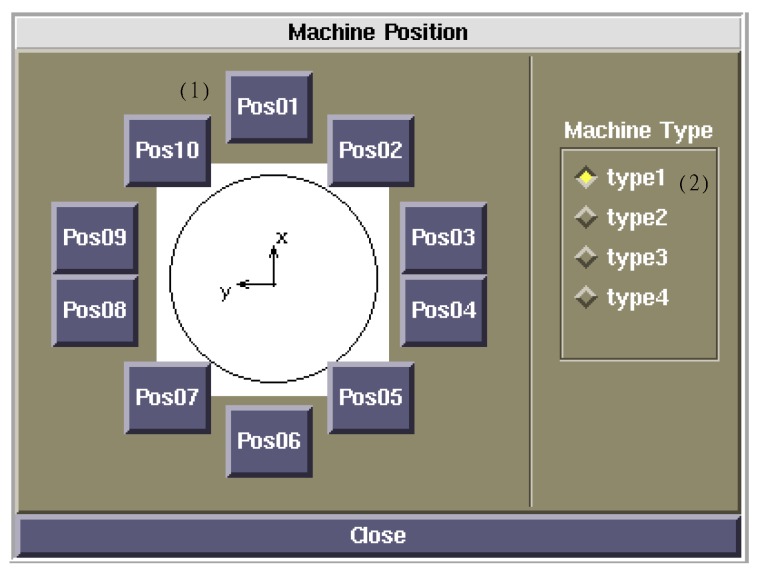
Layout panel. By specifying the four types of microrobots with different weights at each position on the layout panel, the microrobots can be placed arbitrarily. Microrobot type 1: driving microrobot (master), weight: 0.508 g; microrobot type 2: driving microrobot (slave), weight: 0.508 g; microrobot type 3: flaw-detection microrobot (non-driving), weight: 0.318 g; microrobot type 4: transmission microrobot (non-driving), weight: 0.304 g.

In this simulator, the following four types of microrobots were considered according to the prototype microrobot. Microrobot type 1 is a driving microrobot (master) of 0.508 g, microrobot type 2 is a driving microrobot (slave) of 0.508 g, microrobot type 3 is a flaw-detection microrobot (non-driving) of 0.318 g, and microrobot type 4 is a transmission microrobot (non-driving) of 0.304 g. By specifying the four types of microrobots with different weights at each position on the layout panel shown in Figure 4, the microrobots were placed arbitrarily.

#### 2.2.2. Hoop Force Acting on the Microrobot

**Figure 5:**
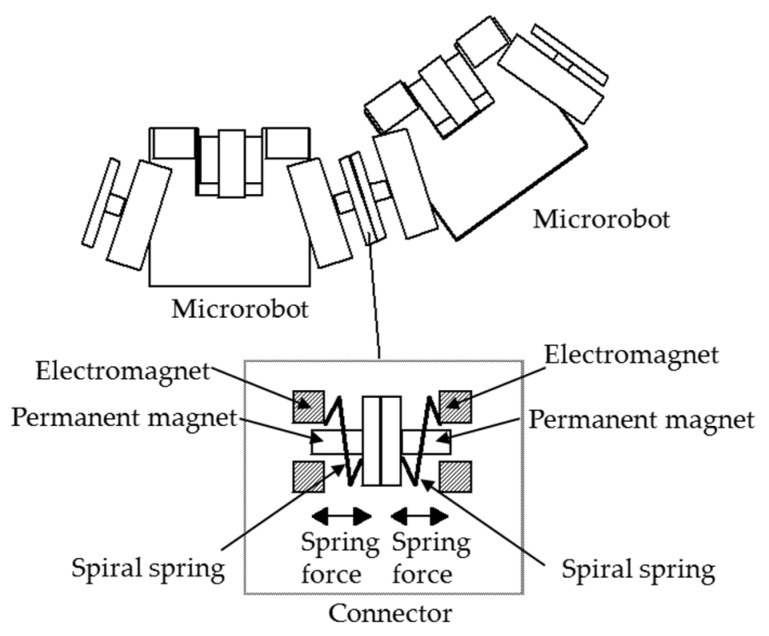
Interaction between microrobots. The spiral spring connected between the microrobot body and the connector device is extended compared to its natural length. The connector can be automatically attached and detached by electromagnets, and after connection, the connection is held by the magnetic attraction force of the permanent magnets arranged inside the connector.

As shown in Figure 5, in the state where the microrobots are connected to each other, the spiral spring connected between the microrobot body and the connector device is extended compared to its natural length. Therefore, a spring force proportional to the spring extension acts on the microrobot as an external force. The direction of the force is perpendicular to the mated connector device. The spring constant for the spring force of the spiral spring in the horizontal direction was set to 1.85 × 10^−2^ N/mm, and it was calculated as a function of elongation. The spring constant was determined from the measurement of the prototype microrobot. The maximum stroke was set to 800 μm. The connector can be automatically attached and detached by electromagnets, and after connection, the connection is held by the magnetic attraction force (1.16 × 10^−2^ N) of the permanent magnets arranged inside the connector.

**Figure 6:**
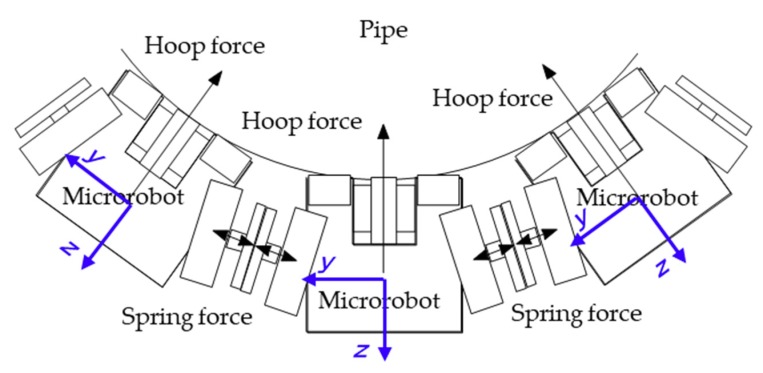
Hoop force acting on the microrobot. The spring force is converted to the microrobot coordinate system and used as an external force in the equation of motion. The hoop force is the force component acting in the negative z direction of the microrobot coordinate system.

The spring force is converted to the microrobot coordinate system and used as an external force in the equation of motion. As shown in Figure 6, the hoop force is the force component acting in the negative *z* direction of the microrobot coordinate system.

#### 2.2.3. Vertical Force Acting between the Microrobots

**Figure 7:**
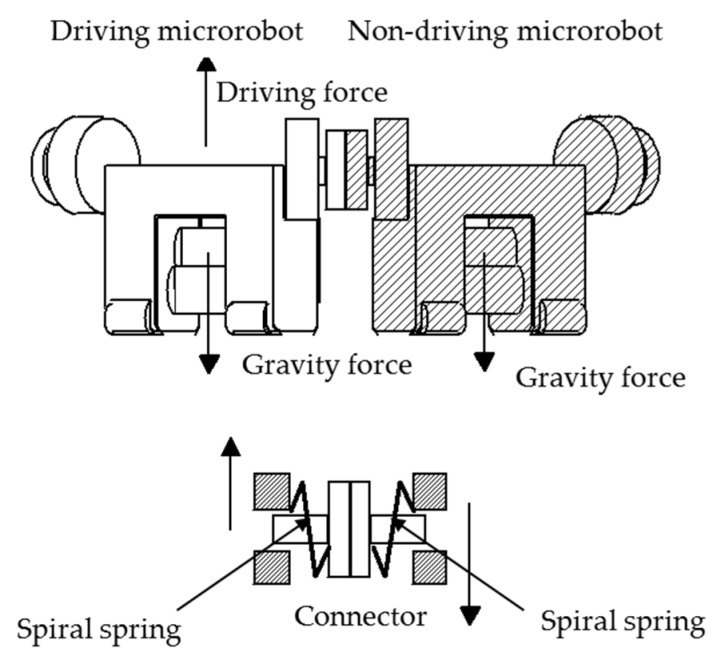
Non-driving microrobot pulled up by the driving microrobot. The non-driving microrobot can climb the small cylinder in the multiple connected state by being pulled up by the driving microrobot. While climbing, the load on the non-driving microrobot is only the gravity load acting in the vertically downward direction. The microrobots are connected through a connector device, and a force acts in the perpendicular direction to the expansion and contraction direction of the spiral spring.

As shown in Figure 7, a non-driving microrobot without the driving device can climb the small cylinder in the multiple connected state by being pulled up by the driving microrobot possessing driving devices. While climbing, the load of the non-driving microrobot without the driving force is only the gravity load acting in the vertically downward direction. The microrobots are connected through a connector device, and a force acts in the perpendicular direction to the expansion and contraction direction of the spiral spring. Therefore, the elevation position of each microrobot may deviate due to the rigidity of the spiral spring in the vertical direction. The spring constant in the vertical direction was set to 5.79 × 10^−2^ N/mm from the measurement of the prototype microrobot.

#### 2.2.4. Multiple Connected Movement

**Figure 8:**
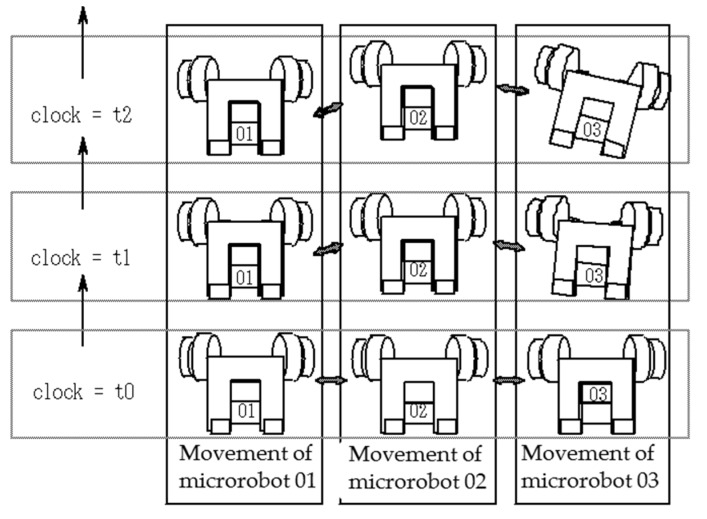
Image of multiple connected movement. First, by focusing on the motion of each individual microrobot, as the simulation time progresses from t0 to t1 to t2, the respective position of each microrobot will change based on the applied rotational speed, acting gravity, and wheel magnetic attraction force.

A diagram of multiple connected movement is shown in Figure 8. First, if we focus on the motion of each individual microrobot, as the simulation time progresses from t0, t1, to t2, the respective position of each microrobot will change based on the applied rotational speed, acting gravity, and wheel magnetic attraction force. At this time, each microrobot maintains the connected state with the action of the magnetic attraction force from adjacent microrobots and the connecter device. The spiral spring is present between the connector device and the microrobot, and the gap between the microrobots changes with the extension of the spring. The interaction from the adjacent microrobots differs at each simulation time in accordance with the position changes of each microrobot. In the vertical climbing simulator for multiple connected microrobots, instead of deriving the solution by establishing the overall equation of motion with the motion of all microrobots as one system, the equation of motion is established in parallel for each microrobot, and the influence of the connected and adjacent microrobots is applied to each as an external force of the equation of motion.

#### 2.2.5. Evaluation and Determination of Connected Movement

The spring force generated between adjacent simulation models is proportional to the distance between the models and acts as an external force on individual models. Accordingly, it is necessary to understand the position of each simulation model on the small cylinder and calculate the spring force from the distance with the adjacent model.

If a model cannot move straight upward due to an impact on the interaction between the adjacent models, the distance between the models must be increased or decreased. In this situation, if the connection is broken due to the generation of a spring force exceeding the magnetic attraction force of the connecter device or if an interference occurs between the models due to the inclination of the simulation model, then connected motion is not possible.

## 3. Simulation Results

### 3.1. Independent Microrobot Movement Simulation Result

#### 3.1.1. Relationship between the Magnetic Attraction Force of Wheels and Vertical Climbing Capability

**Figure 9:**
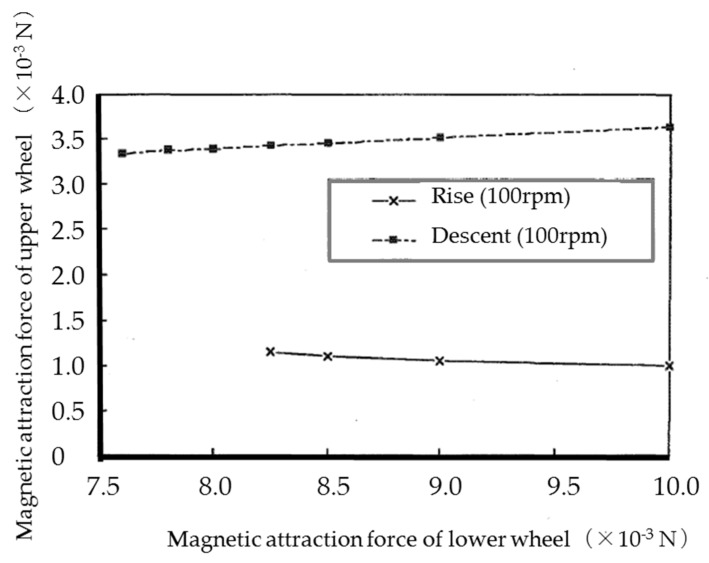
The relationship between the magnetic attraction force of the upper and lower wheels of a single microrobot capable of vertical movement up and down. We observed that a larger magnetic attraction force is required for the upper wheels when descending than when climbing. It is better if the magnetic attraction force of the lower wheels is larger and the magnetic attraction force of the upper wheel is smaller when climbing, and the opposite is true for descending.

The relationship between the magnetic attraction force of the upper and lower wheels of a single microrobot capable of vertical movement up and down at a friction coefficient of 0.3 is shown in Figure 9. Figure 9 shows that a larger magnetic attraction force is required for the upper wheels when descending than when climbing. It is better if the magnetic attraction force of the lower wheels is larger and the magnetic attraction force of the upper wheels is smaller when climbing, and the opposite is true when descending. When descending, since the separation moment of the upper wheels increases due to the downward acceleration, a magnetic attraction force must be applied on the upper wheels to overcome this. Vertical climbing was not possible unless a magnetic attraction force of 8.25 × 10^−3^ N or more per lower wheel and 1.15 × 10^−3^ N or more per upper wheel was applied when climbing, and 7.6 × 10^−3^ N or more per lower wheel and 3.4 × 10^−3^ N or more per upper wheel when descending.

#### 3.1.2. Relationship between the Magnetic Attraction Force of the Lower Wheels and the Slip Ratio with Climbing Velocity

**Figure 10:**
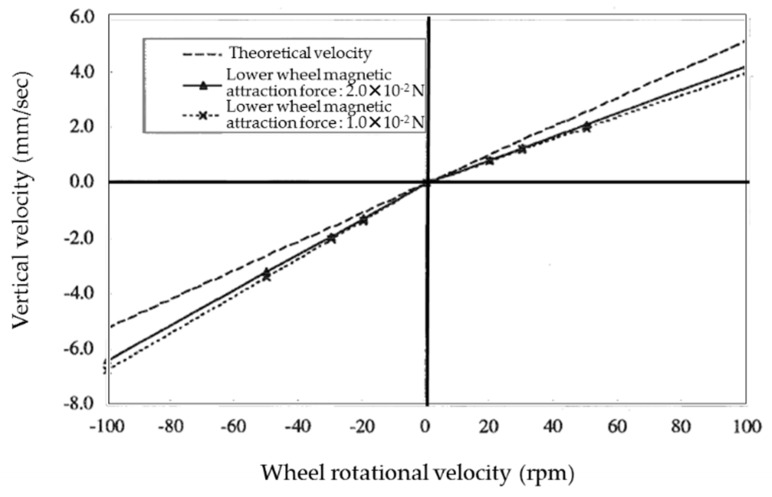
Relationship between the vertical velocity and wheel rotational velocity. Due to the impact of gravity, the observed vertical velocity was lower than the theoretical velocity when climbing and higher than the theoretical velocity when descending.

Figure 10 shows the relationship between the climbing velocity and wheel rotational velocity when the magnetic attraction force per wheel is 5.0 × 10^−3^ N for the upper wheels and the magnetic attraction force used for the lower wheels is 1.0 × 10^−2^ N and 2.0 × 10^−2^ N at a friction coefficient of 0.3. Due to the impact of gravity, the observed climbing velocity is lower than the theoretical velocity when climbing and higher than the theoretical velocity when descending. Although the slope of the climbing velocity with respect to the wheel rotational velocity differs between climbing and descending, it is constant and independent of the wheel rotational velocity. Slip can also be considered to be constant and independent of the wheel rotational velocity. The velocity is observed to approach the theoretical value when the magnetic attraction force of the lower wheels is increased, but by a small degree.

**Figure 11:**
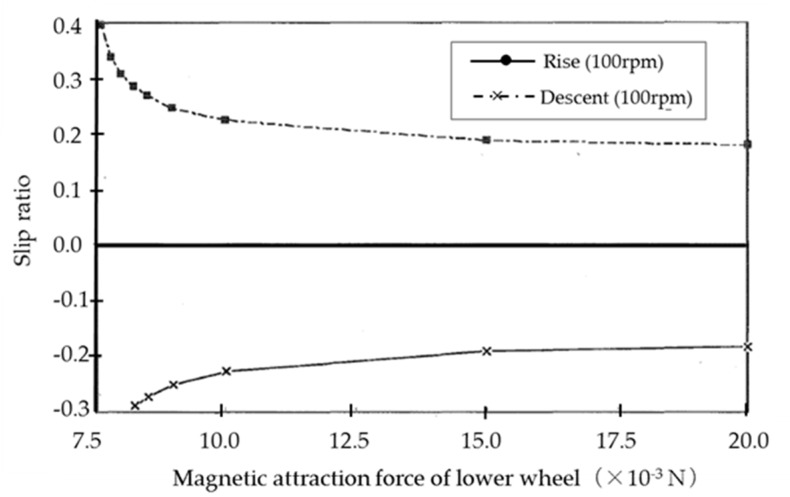
Relationship between the slip ratio and the magnetic attraction force of the lower wheels when climbing and descending at 100 rpm. For both climbing and descending, the slip ratio decreases as the gripping force increases with the increase in the magnetic attraction force of the lower wheels. Since the slip ratio was observed to converge to a particular value, the climbing velocity converges not to the theoretical velocity.

Figure 11 shows the relationship between the slip ratio and the magnetic attraction force of the lower wheels when climbing and descending at 100 rpm. The slip ratio ($s_{wi}$) of wheel wi is calculated as follows:

(3) $$s_{wi}=\frac{u_{wi}-{\bar{r}}_{wi}\times {\dot{\emptyset }}_{wi}}{u_{wi}},$$

wher$e\;{\bar{r}}_{wi}$ is the radius of wheel *i*,$\;u_{wi}$ is the x-axis directional speed of wheel *i*,$\;\mathrm{and}\;{\dot{\emptyset }}_{wi}$ is the rotating speed of wheel *i*.

Though we observed that the velocity due to the rotation of the wheels was even higher than the microrobot velocity and the slip ratio was a negative value when climbing, the opposite held true when descending. For both climbing and descending, the slip ratio decreased as the gripping force increased with the increase in the magnetic attraction force of the lower wheels. Since the slip ratio was observed to converge to a particular value, the climbing velocity could not converge to the theoretical velocity.

### 3.2. Simulation Results of the Movement of Multiple Connected Microrobots

**Figure 12:**
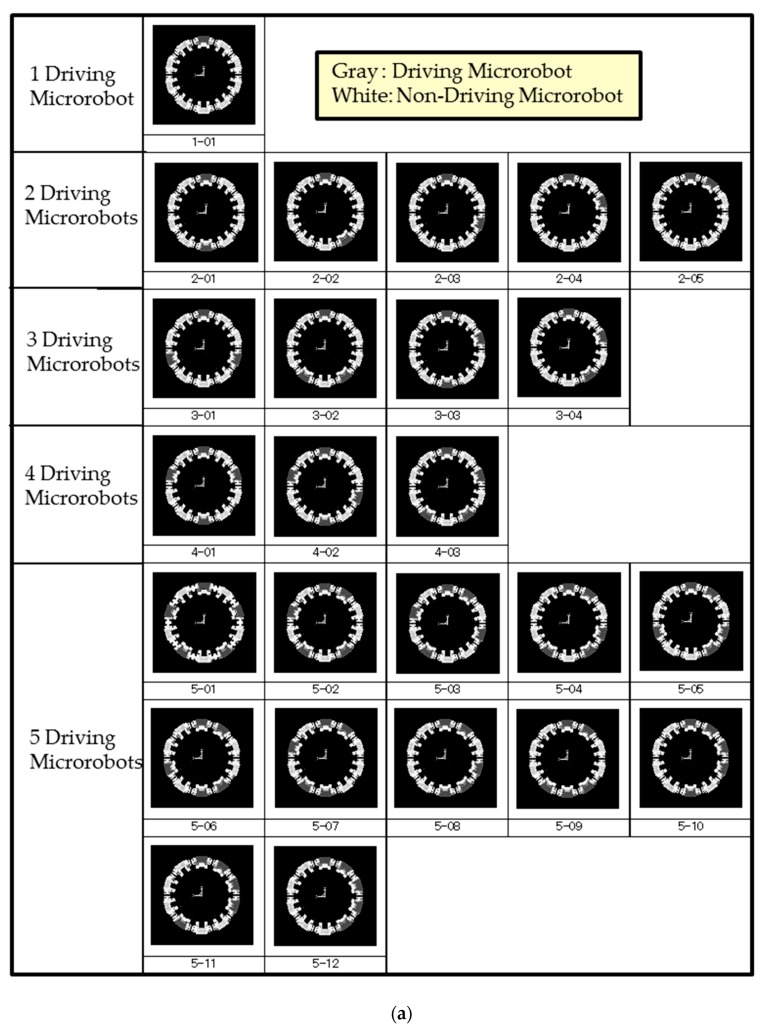

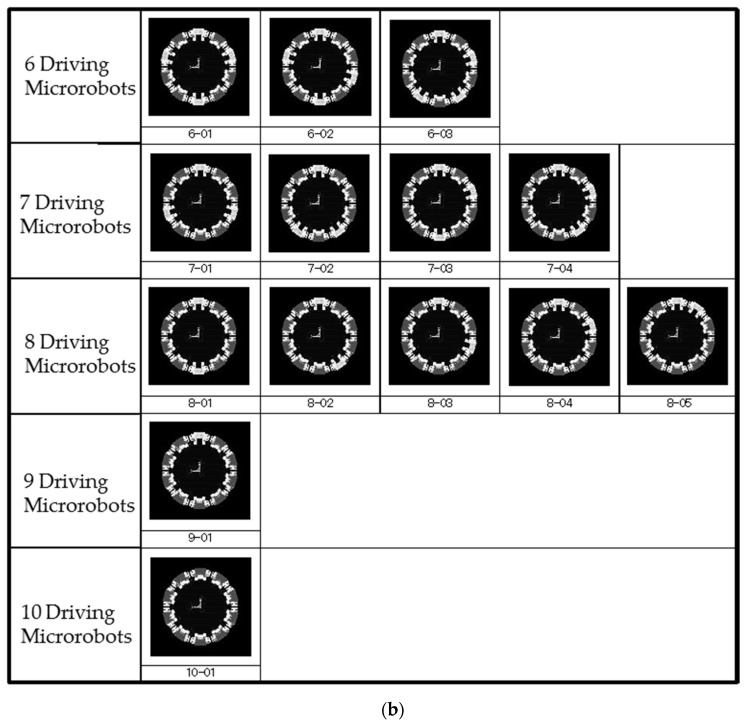
Layout patterns when surrounding the pipe with 10 microrobots. All combinations of driving microrobots and non-driving microrobots when surrounding the pipe with 10 microrobots are shown. Gray microrobots are the driving microrobots and white microrobots are the non-driving microrobots. (**a**) Layout patterns for 1 to 5 driving microrobots and (**b**) layout patterns for 6 to 10 driving microrobots.

All combinations of driving microrobots and non-driving microrobots surrounding the pipe with 10 microrobots are shown in Figure 12a,b. 

In each arrangement, a one-second rise simulation was performed in time intervals of 0.001 s. The average rise height value of the 10 machines, the rise height SD, the lateral SD, and the rotational SD were calculated, respectively. The calculated results are shown in Table 1.

**Figure 13:**
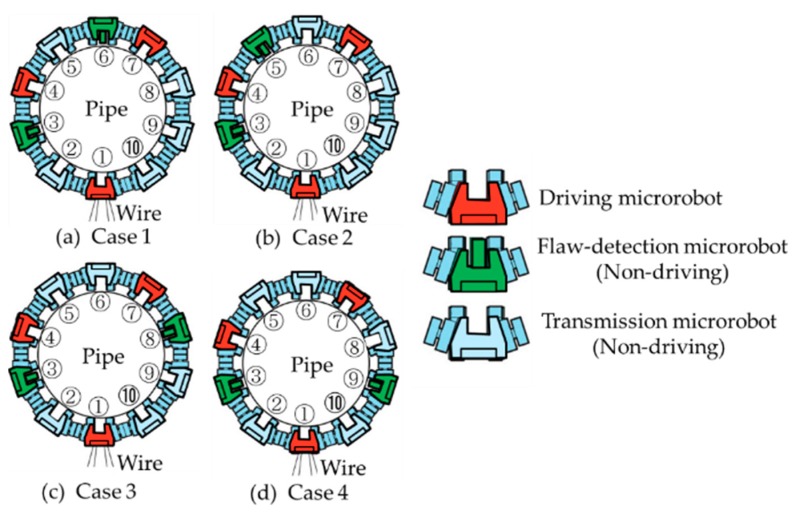
Ten microrobots were arranged around a pipe. There were three types of microrobots: a driving microrobot that has driving devices, a flaw-detection microrobot that has a flaw-detection sensor but does not have driving devices, and a transmission microrobot that transmits power and sensor signals but does not have driving devices. Four cases are shown, in which the positions of the two flaw-detection microrobots are different. In case 1 and case 2, the flaw-detection microrobots were placed between the driving microrobots ① and ④, and ④ and ⑦. In case 1, the flaw-detection microrobots were placed on the right side of the driving microrobots ④ and ⑦. In case 2, the flaw-detection microrobots were placed on both sides of the driving microrobot ④. In case 3 and case 4, the flaw-detection microrobots were placed between the driving microrobots ① and ④, and ① and ⑦. The difference between cases 3 and 4 was the presence or absence of the driving microrobot next to the flaw-detection microrobot between the driving microrobots ① and ⑦.

Table 1 shows that three driving microrobots are necessary for pipe climbing. To evaluate the influence of the adjacent microrobots, we examined the case where there are only three driving microrobots, which has the largest influence from adjacent microrobots. As an example for the movement of multiple connected microrobots, 3 out of 10 microrobots are driving microrobots with driving devices and the other 7 microrobots are non-driving microrobots, of which 2 are flaw-detection microrobots for detecting flaws in pipes, and the remaining 5 are transmission microrobots having only power and signal transmission functions. As the driving microrobots, flaw-detection microrobots, and transmission microrobots have different weights, we examined whether the movement of 10 connected microrobots would be possible by arranging each microrobot around a pipe through simulation. Simulations were performed for the four cases shown in Figure 13 to compare the deflection of each microrobot with the current spring rigidity and at a friction coefficient of 0.3 to observe the effect of arranging two flaw-detection microrobots when three driving microrobots were arranged at intervals of two, two, and three microrobots. The mass of each microrobot obtained by measurement was 0.508 g for the driving microrobot, 0.318 g for the flaw-detection microrobot, and 0.304 g for the transmission microrobot. The driving device rotational velocity of the driving microrobots was set to 20 rpm, the coefficient of friction to 0.3, and the magnetic attraction force of the driving wheels to 4.0 × 10^−2^ N, which was sufficiently large. The spring constant of the spiral spring was 1.85 × 10^−2^ N/mm and was 5.79 × 10^−2^ N/mm in the vertical direction.

**Figure 14:**
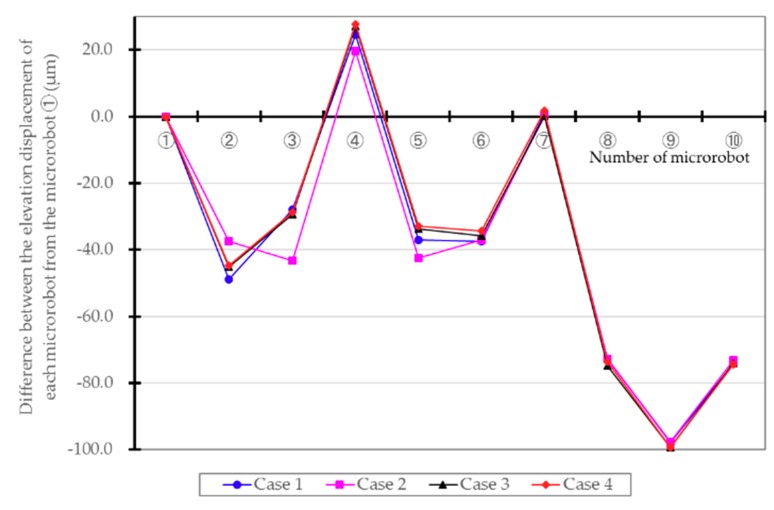
Difference between the elevation displacement of each microrobot from the parent microrobot ①. The other microrobots are being pulled up by microrobots ①, ④, and ⑦, which are the driving microrobots. The gaps between microrobots ⑦ and ⑧ and between microrobots ⑩ and ① were the largest, and the maximum was approximately 75 μm.

**Figure 15:**
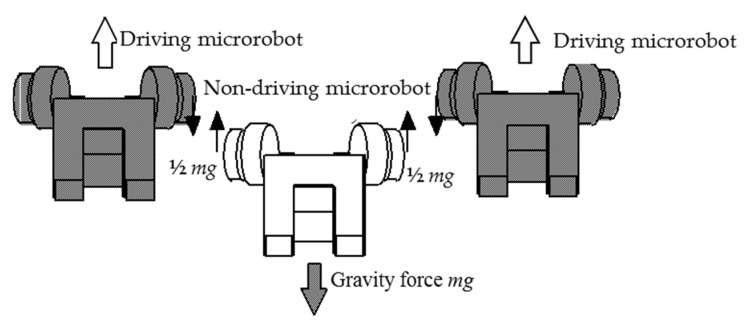
Image of non-driving microrobot pulled up by driving microrobot. *m*g is the gravity force.

The difference in the climbing displacement (μm) of each microrobot, with the elevation position of the parent microrobot (driving microrobot ①) having a power line as reference, is shown in Table 2 and Figure 14 for each case. These values are the average calculated values for 10 sets of data collected every 0.1 s. In each case, as shown in Figure 15, the other non-driving microrobots are being pulled up by microrobots ①, ④, and ⑦, which are the driving microrobots. Since the mass of the flaw-detection microrobot is slightly greater than that of the transmission microrobot, there were some differences in climbing displacement in each case. In case 2, since the flaw-detection microrobots are present on both sides of microrobot ④, the elevation of microrobot ④ is lower than in the other cases. Conversely, in cases 3 and 4, where the load on microrobot ④ is smaller, the elevation position of microrobot ④ is higher, resulting in a wider difference in displacement between the microrobots. When the difference between the relative elevation positions of each microrobot was determined, the gaps between microrobot ⑦ and ⑧ and between microrobot ⑩ and ① were the largest, and the maximum was approximately 75 μm. The tolerance between the connector and the electromagnetic coil was less than 100 μm, and the connector did not come in contact with the electromagnetic coil during connected movement of the microrobots.

**Figure 16:**
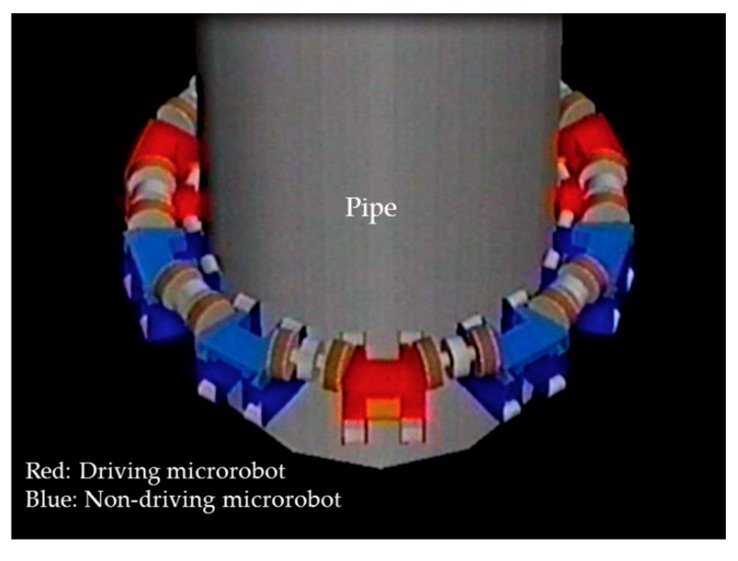
Simulation results (animation display) of 10 connected microrobots. Red microrobots show driving microrobots and blue microrobots show non-driving microrobots. The animation result shows that three driving microrobots can move seven non-driving microrobots.

We found that three driving microrobots could move seven non-driving microrobots comprising 60% of their own weight up and down along a vertical pipe. An example of the animation display results of the connected movement simulation for 10 microrobots is shown in Figure 16.

## 4. Prototype and Performance Evaluation of Pipe-Climbing by the Magnetic Wheel-Driven Microrobot

### 4.1. Configuration of Microrobot Prototype

**Figure 17:**
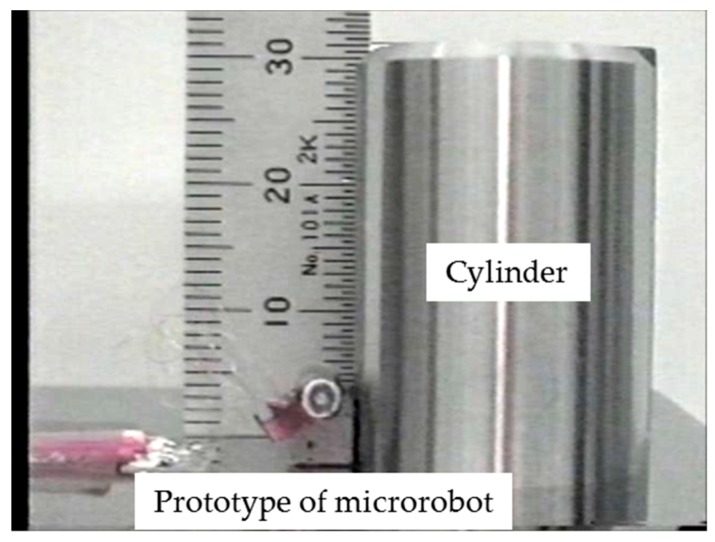
Prototype of a microrobot for evaluating the independent vertical driving performance of a microrobot. A cylinder made of magnetic stainless steel (SUS 430) with a diameter of 22 mm and a height of 30 mm was used in the experiment as a target object simulating a pipe.

**Figure 18:**
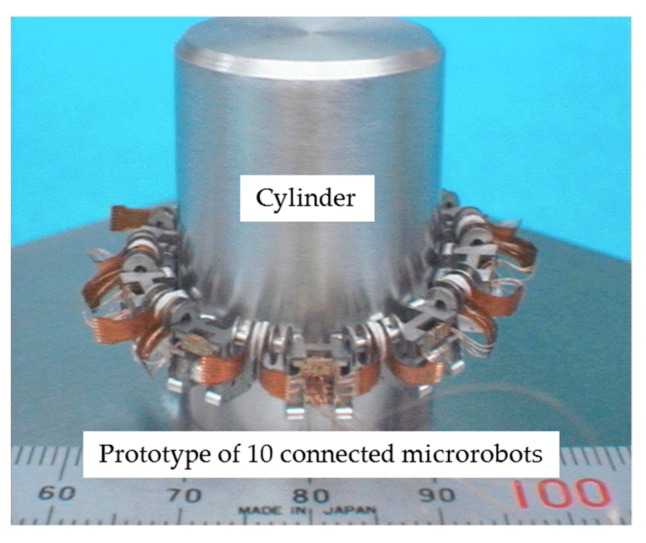
Prototype of 10 connected microrobots for evaluating the vertical driving performance with 10 connected microrobots. Ten microrobots were arranged around a cylinder simulating a pipe. There were three types of microrobots: a driving microrobot with driving devices, a flaw-detection microrobot with a flaw-detection sensor but no driving device, and a transmission microrobot with transmit power and sensor signals but no driving device.

We manufactured prototypes of an independent vertical climbing microrobot (Figure 17) to evaluate the independent vertical driving performance and of 10 connected microrobots (Figure 18) to evaluate the vertical driving performance with 10 connected microrobots. A cylinder made of magnetic stainless steel (SUS 430) with a diameter of 22 mm and a height of 30 mm was used in the experiment as a target object simulating a pipe.

**Figure 19:**
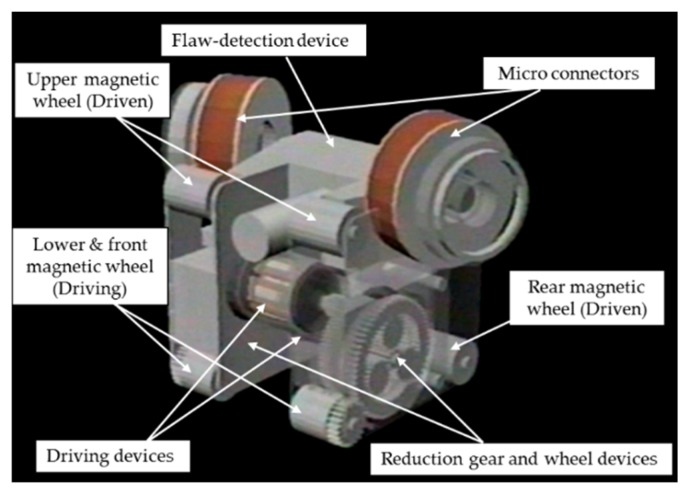
Configuration of a prototype of a magnetic wheeled microrobot with four types of functional devices: driving devices, reduction gear and wheel devices, micro connectors, and a flaw-detection device.

The configuration of the prototype microrobot is shown in Figure 19. The microrobot is composed of four types of functional device: driving devices, reduction gear and wheel devices, micro connectors, and a flaw-detection device. As shown in Figure 19, assembly was facilitated with a simple structure in which the reduction gear and wheel devices were arranged on the right and left sides of the two driving devices and the flaw-detection device was mounted with micro connectors arranged above on the right and left sides. A magnetic wheel with a 1 mm diameter was adopted, and a magnetic wheel structure with six wheels was used, which was capable of climbing vertical surfaces, as shown in Figure 19. The microrobot size was 5 mm long, 9 mm wide, and 6.5 mm high, and the weight of the driving microrobot was 0.508 g, that of the flaw-detection microrobot was 0.318 g, and that of the transmission microrobot was 0.304 g. The magnetic attraction force of the lower (front wheels) driving wheels was 7.1 × 10^−2^ N, and the magnetic attraction force of the upper driven wheels was 1.1 × 10^−2^ N. The microrobot was driven as follows. The same rotational speed was commanded to the two driving devices from the microrobot controller. The driving device rotated to the target speed according to the command. The rotation of the two driving devices was transmitted to the left and right reduction gear and wheel devices. In the reduction gear and wheel devices, the rotation of the driving device was decelerated to 1/200 by the planetary gear reducer and transmitted to the lower magnet wheel. The microrobot moved the cylinder up and down as the lower magnet wheel rotated.

### 4.2. Microrobot Vertical Driving Performance Evaluation and Control System

**Figure 20:**
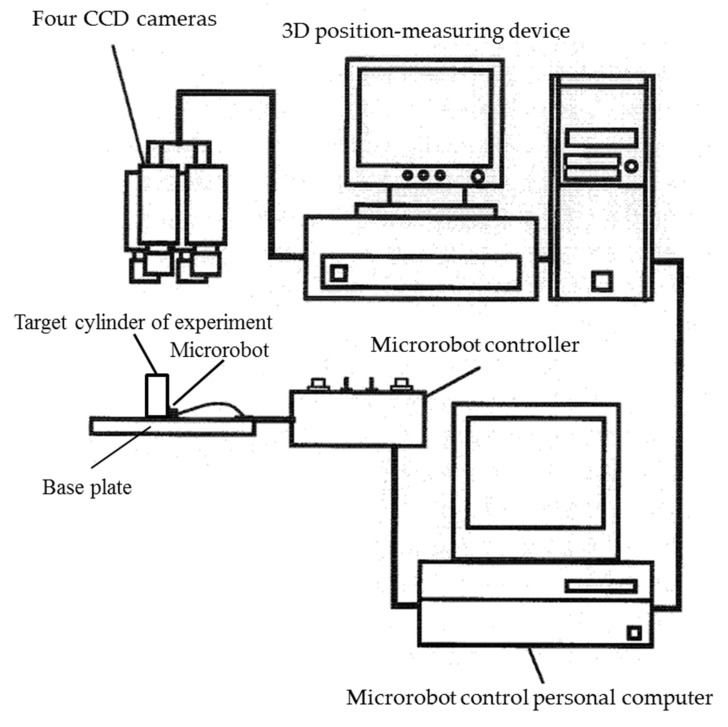
Microrobot vertical driving performance evaluation and control system. The system consisted of six parts: (1) 4 CCD cameras to capture images of the microrobots, (2) a 3D position-measuring device to extract the three color marks from the images and measure the position and orientation of the microrobots, (3) a computer that outputs a control signal to control the microrobot by executing the control program based on the position information, (4) a microrobot controller to drive the driving device of the microrobot after receiving the control signal, (5) the microrobot that is controlled, and (6) the target cylinder simulating a pipe on a base plate.

The microrobot vertical driving performance evaluation and control system is shown in Figure 20. The system consisted of six parts: (1) four CCD (Charge-Coupled Device) cameras to capture images of the microrobots, (2) a 3D position-measuring device to extract the three color marks from the images and measure the position and orientation of the microrobots, (3) a computer that outputs a control signal to control the microrobot by executing the control program based on the position ∆information, (4) a microrobot controller to drive the driving device of the microrobot after receiving the control signal, (5) the microrobot that was controlled, and (6) a target cylinder simulating a pipe on a base plate. The CCDcamera uses a color 3CCD camera (TN411, made by ELMO, Nagoya, Japan) to recognize the color marks, and the resolution was 768 × 494 pixels. The lens used was 25 mm F1.4 (compact ITV (Industrial television) lens made by the Sakai Glass Co., Ltd., Osaka, Japan). The 3D position-measuring device consisted of an image recognition device (Quick/MAGAV, made by OKK, Itami, Japan) and data processing computer (Endeavor Pro400, made by EPSON DIRECT, Matsumoto, Japan), and images were captured at a frame rate of 60 frames/s. A memory link was used to connect the data processing computer and the computer (PC9821, made by NEC, Tokyo, Japan) connected to the microrobot, and data were exchanged in real time. We manufactured the microrobot controller, which had manual and automatic modes, and the control was performed using the control computer in automatic mode. Since the color marks were small, with a diameter of 1 mm, for stable measurement of the marks, auxiliary illumination was used along with shielding of ambient light. Magnetic SUS 430 of an approximate diameter of 22 mm, height of 30 mm, and surface roughness of 1 µm R_max_ (peak-to-peak roughness) was used as the cylinder simulating a pipe in the experiments.

### 4.3. Vertical Driving Performance Measurement Results

#### 4.3.1. Measurement Method

The Supplementary Video S1 shows how single microrobot moves up and down the cylinder wall. The movement when climbing and descending the cylinder wall was measured using the microrobot vertical driving performance evaluation and control system shown in Figure 20. To vertically climb and descend, the rotation speeds of the left and right drive devices were identical. Three marks were used for evaluation, and the 3D position and posture were measured. The movements when climbing and descending were measured by setting the drive voltage of the driving device to three different voltages—0.5 V, 0.7 V, and 0.9 V—by gradually changing the rotational speed of the driving device (1200 rpm to 12,000 rpm, corresponding to a rotational speed of 6 rpm to 60 rpm of the driving wheel).

#### 4.3.2. Measurement Results

**Figure 21:**
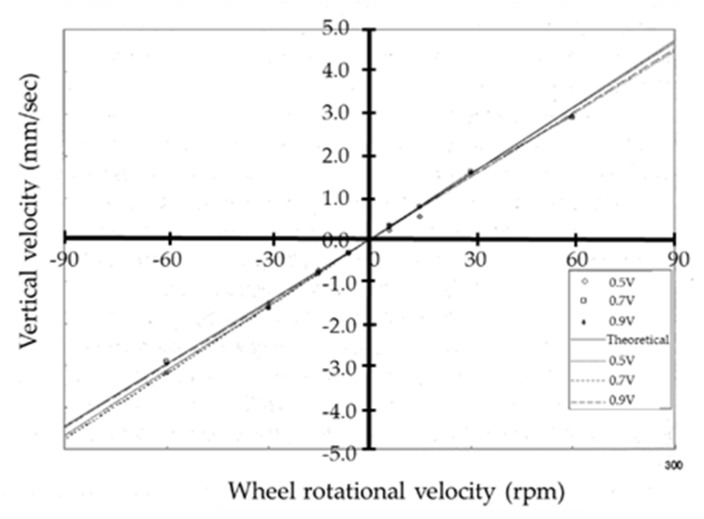
The relationship between the wheel rotational velocity and climbing velocity in the vertical direction obtained from the measurement results. Descending movement is indicated by negative wheel rotational velocity values, the straight line is the theoretical value of the wheel rotational velocity, and the dashed lines are the result of approximating the measurement results, using the least squares method, to straight lines passing through the origin.

The relationship between the wheel rotational velocity and the climbing velocity in the vertical direction obtained from the measurement results is shown in Figure 21. In the figure, descending movement is indicated by negative wheel rotational velocity values, the straight line is the theoretical value of the wheel rotational velocity, and the dashed lines are the result of approximating the measurement results, using the least squares method, to straight lines passing through the origin. The straight-line approximation is not shown for a drive voltage of 0.5 V as the device could not ascend at 30 rpm for this voltage. From Figure 21, for the 0.5 V drive voltage, the travel velocity is slower than the theoretical value determined for the wheel rotational velocity when climbing, faster when descending, and there is almost no difference between the measured and calculated values for the driving voltages of 0.7 V and 0.9 V, with the velocity close to the theoretical value of the wheel rotational velocity for both climbing and descending, and driving performance necessary and sufficient for vertical climbing was produced. The velocity at the drive voltage of 0.5 V did not match the theoretical velocity because the necessary torque required for climbing could not be achieved when the drive voltage was low, as the driving device repeated an instantaneous rotation and stopped the process, reducing the apparent rotational velocity. In addition, the weight of the microrobot could not be supported by the driving device when descending, causing an increase in the rotation of the driving device and increasing the apparent rotational velocity.

### 4.4. Connected Microrobot Vertical Driving Performance Evaluation Results

#### 4.4.1. Measurement Method

**Figure 22:**
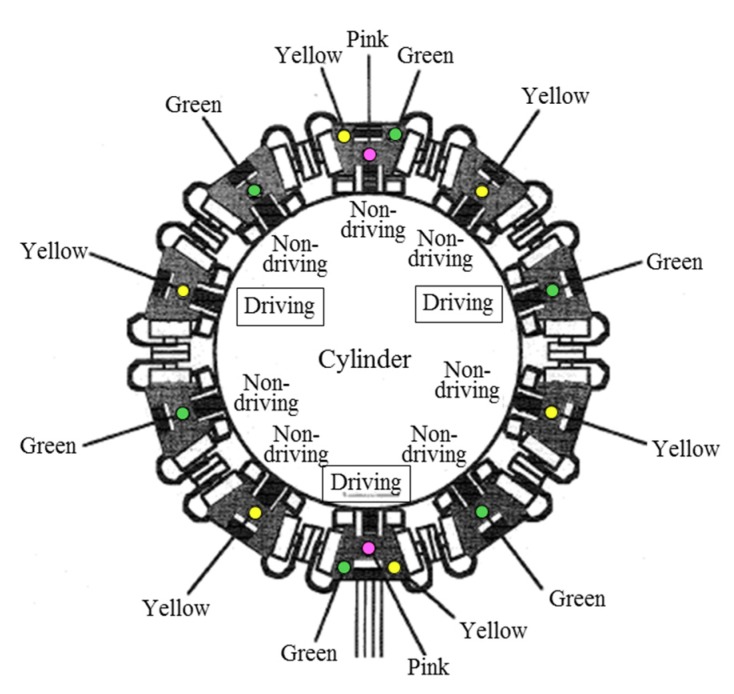
The layout of 10 connected microrobots and the arrangement of the marks for measurement. Among the 10 connected microrobots, the driving microrobot consisted of 3 microrobots: 1 main microrobot receiving the power supply by wire and 2 sub-microrobots receiving the power supply from the main microrobot through the connector; both are shown enclosed in a box. Three marks were illustrated in the main microrobot and the opposite microrobot. One mark each was illustrated for the other eight microrobots, and a different colored mark was used for the adjacent microrobot.

The microrobot vertical driving performance evaluation and control system shown in Figure 20 were used to examine the vertical driving performance when 10 microrobots connected using connectors climb and descend the cylinder wall. Figure 22 shows the layout of the 10 connected microrobots and the arrangement of the marks for measurement.

As shown in Figure 22, among the 10 connected microrobots, the driving microrobot capable of movement that was embedded with the driving device and reduction gear and wheel devices consisted of three microrobots: one main microrobot receiving the power supply by wire and two sub-microrobots receiving the power supply from the main microrobot through the connector; both are shown enclosed in a box. Three marks were illustrated in the main microrobot and the opposite microrobot. The reason for illustrating the three marks in the two microrobots was to examine the change in posture of the microrobots while climbing. One mark each was illustrated for the other eight microrobots, and a different color was used for the adjacent microrobot. Different colored marks were selected for the sub-microrobots so that the large amount of data received by the 3D position-measuring device could be processed efficiently. The experiment was conducted by setting the frequency of the power supply to the main microrobot and sub-microrobots to 106 Hz (approximately 32 rpm when converted to the rotational velocity of the magnetic wheels).

#### 4.4.2. Measurement Results

**Figure 23:**
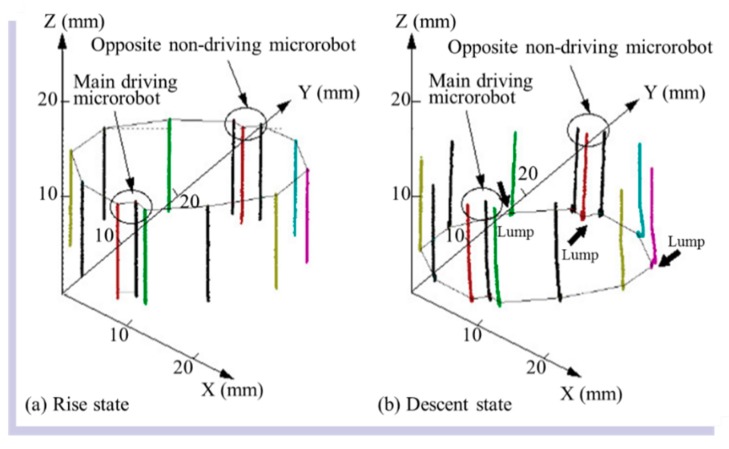
Path of the marks on the microrobots during vertical (**a**) climbing and (**b**) descending by the connected microrobots. The path spread at the points indicated by the thick arrows in (**b**) because the microrobots were being driven even after reaching the base plate, and the wheels slipped on the base plate, causing the microrobots to vibrate.

The Supplementary Video S2 shows how 10 connected microrobots move up and down the cylinder wall. Figure 23 provides an example of the measurement results, showing the path of the marks of the 10 connected microrobots during vertical climbing. Figure 23a shows the climbing path, and Figure 23b shows the descending path. Figure 23 shows that it was possible to measure the position change during the movement of the 10 connected microrobots. The path spread at the points indicated by the thick arrows in Figure 23b because the microrobots were being driven even after reaching the base plate, and the wheels slipped on the base plate, causing the microrobots to vibrate.

**Figure 24:**
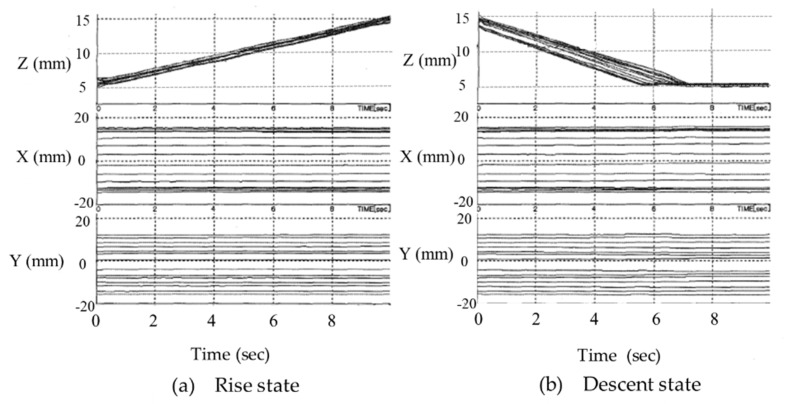
Measurement results of the position of the 10 microrobots when (**a**) rising and (**b**) descending. The *X*-axis represents the tangential direction, the *Y*-axis represents the radial direction, and the *Z*-axis represents the axial direction of the cylinder. There was almost no change in the in-plane position (X and Y directions) on the base plate, for both climbing and descending. From (**b**), the position of the Z-direction does not change after approximately six seconds when descending.

Based on the results shown in Figure 23, changes in the position and velocity were examined for the vertical climbing of the connected microrobots. The positions of the 10 microrobots when climbing and descending are shown in Figure 24a,b, respectively. When Figure 24a,b is compared, almost no change in the in-plane position (X and Y directions) on the base plate can be observed for both climbing and descending. Figure 24b shows that the position in the *Z* direction does not change after approximately six seconds when descending. From this, we found that the microrobot reached the lower surface, which is the base plate.

**Figure 25:**
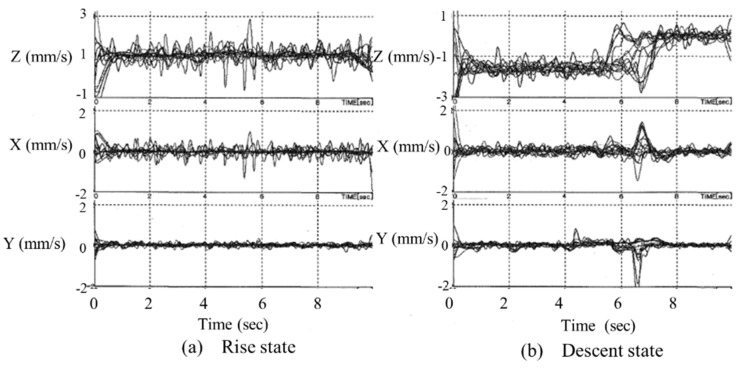
Measurement results of the velocity of 10 microrobots in the (**a**) climbing and (**b**) descending states. The velocity in the *Z*-direction when descending is twice that when climbing. The velocity change in the *Y*-direction is small compared to the other two directions as the movement of the microrobots is restricted by the cylinder wall. The velocity fluctuation in the three directions is high in the time range between 0 and 1 s.

Figure 25a,b shows the velocity of each microrobot when climbing and descending, respectively. When Figure 25a,b is compared, the velocity in the Z-direction when descending is twice the velocity when climbing and matched the measured positions of the microrobots as shown in Figure 24. In other words, when descending, the velocity of the microrobots being higher compared to when climbing indicates that the microrobot reaches the base plate surface in a short time and that the position of the microrobot does not change after approximately six seconds. In Figure 25b, the velocity fluctuates significantly after approximately six seconds in the X, Y, and Z directions when descending because the microrobots vibrate upon reaching the base plate. The velocity change in the *Y*-direction is small compared to the other two directions as the movement of the microrobots is restricted by the cylinder wall. Figure 25a,b shows that the velocity fluctuation in the three directions is high from 0 to 1 s. The beginning of movement is unstable because of the need to pull up and down the stationary non-driving microrobot. In addition, when the velocity in each direction from 0 to 6 s is compared, the fluctuation rate of the velocity is higher when climbing than when descending. The microrobot is more likely to tilt when climbing than when descending because of the increased load to lift the seven non-driving microrobots that do not have power. 

## 5. Conclusions

Based on the analysis of the dynamics of magnetic wheel-driven microrobots considering the magnetic attraction force and slip during pipe climbing, we determined the condition of the magnetic attraction force necessary for pipe climbing, and we measured the vertical driving performance of an independent microrobot and 10 connected microrobots via simulation and the creation of prototypes of the microrobots. The following conclusions were obtained.

The magnetic attraction force required per wheel by the upper and lower wheels of a microrobot are as follows: for vertical climbing, lower wheels 8.25 × 10^−3^ N and upper wheels 1.15 × 10^−3^ N; for descending, lower wheels 7.6 × 10^−3^ N and upper wheels 3.7 × 10^−3^ N.

We examined the movement of multiple connected microrobots, which has been poorly studied until now. A climbing simulator for multiple connected microrobots was developed to simulate the situation where 10 connected microrobots are located around a small cylinder and move up and down, clarifying the conditions for multiple connection movement. Using the specifications of the prototype microrobots, we conducted a simulation considering the hoop force and the load from the adjacent microrobots. As a result, we confirmed that seven non-driving microrobots, comprising 60% of the weight of the driving microrobots, could move up and down around the cylinder using three driving microrobots.

To evaluate the influence of adjacent microrobots, we examined a case with only three driving microrobots, which shows the largest influence from adjacent microrobots. The vertical climbing performance of an independent microrobot and 10 connected microrobots using a prototype of the microrobots was evaluated, and the usefulness was clarified by showing that three driving microrobots can move seven non-driving microrobots comprising 60% of their own weight up and down along a small cylinder.

## Figures and Tables

**Table 1:** Simulation results of 10 connected microrobots’ movements surrounding a pipe. Layout No. indicates the same numbers shown in Figure 12. In the yellow background case, the robot is disconnected, and the pipe cannot be climbed.

Layout No.	Rise Height Average Value (mm)	Rise Height Standard Deviation (mm)	Lateral Standard Deviation (mm)	Rotational Standard Deviation (deg)	Remarks
1-01	0.41	0.6	0.05	2.22	Disconnected
2-01	1.21	0.6	0.08	3.34	Disconnected
2-02	1.03	0.68	0.06	2.72	Disconnected
2-03	1.15	0.91	0.08	2.9	Disconnected
2-04	1.09	1.05	0.09	3.21	Disconnected
2-05	0.82	0.96	0.09	3.17	Disconnected
3-01	9.13	0.42	0.07	2.67	
3-02	8.94	0.5	0.06	3.16	
3-03	8.02	1.22	0.23	4.89	
3-04	2.61	1.76	0.24	6.29	Disconnected
4-01	9.54	0.25	0.04	1.58	
4-02	9.52	0.23	0.02	1.63	
4-03	9.34	0.59	0.11	2.64	
5-01	10.02	0.09	0	0	
5-02	9.94	0.21	0.04	1.1	
5-03	9.95	0.21	0.04	1.04	
5-04	9.71	0.52	0.14	2.36	
5-05	9.86	0.26	0.05	1.44	
5-06	9.86	0.25	0.04	1.56	
5-07	9.72	0.53	0.13	2.37	
5-08	9.23	1.04	0.28	4.07	
5-09	9.63	0.5	0.1	2.53	
5-10	9.24	1.05	0.28	4	
5-11	9.65	0.51	0.12	2.37	
5-12	1.74	1.11	0.11	3.52	Disconnected
6-01	10.06	0.09	0.01	0.5	
6-02	10.06	0.09	0.01	0.5	
6-03	10.06	0.09	0.01	0.5	
7-01	10.11	0.09	0.01	0.63	
7-02	10.11	0.1	0.01	0.54	
7-03	10.11	0.1	0.02	0.53	
7-04	10.11	0.11	0.02	0.4	
8-01	10.15	0.09	0.01	0.55	
8-02	10.15	0.09	0.02	0.53	
8-03	10.15	0.1	0.02	0.4	
8-04	10.15	0.1	0.02	0.4	
8-05	10.06	0.27	0.06	1.24	
9-01	10.19	0.08	0.01	0.39	
10-01	10.23	0	0	0	

**Table 2:** Difference between the elevation displacement of each microrobot from the parent microrobot ①.

	①	②	③	④	⑤	⑥	⑦	⑧	⑨	⑩
Case 1	0.0	−48.9	−28.0	24.7	−37.0	−37.5	0.9	−72.8	−97.8	−74.1
Case 2	0.0	−37.5	−43.3	19.7	−42.4	−37.1	0.4	−72.7	−97.7	−73.2
Case 3	0.0	−45.1	−29.3	27.0	−33.7	−35.7	0.2	−74.9	−99.2	−74.0
Case 4	0.0	−44.8	−28.8	27.7	−32.9	−34.3	1.7	−73.5	−99.3	−74.3

## Supplementary Materials

The following are available online at https://www.mdpi.com/2072-666X/10/8/524/s1, Video S1: Demonstration of pipe climbing of single microrobot, Video S2: Demonstration of pipe climbing of 10 connected microrobots.
